# Auditory and Visual External Cues Have Different Effects on Spatial but Similar Effects on Temporal Measures of Gait Variability

**DOI:** 10.3389/fphys.2020.00067

**Published:** 2020-02-11

**Authors:** Joao R. Vaz, Troy Rand, Jessica Fujan-Hansen, Mukul Mukherjee, Nick Stergiou

**Affiliations:** ^1^Department of Biomechanics, University of Nebraska Omaha, Omaha, NE, United States; ^2^CIPER, Faculty of Human Kinetics, University of Lisbon, Lisbon, Portugal; ^3^The Paley Institute, West Palm Beach, FL, United States; ^4^Department of Environmental, Agricultural and Occupational Health, University of Nebraska Medical Center, Omaha, NE, United States

**Keywords:** walking, fractals, stability, metronomes, biomechanics

## Abstract

Walking synchronized to external cues is a common practice in clinical settings. Several research studies showed that this popular gait rehabilitation tool alters gait variability. There is also recent evidence which suggests that alterations in the temporal structure of the external cues could restore gait variability at healthy levels. It is unknown, however, if such alterations produce similar effects if the cueing modalities used are different; visual or auditory. The modality could affect gait variability differentially, since there is evidence that auditory cues mostly act in the temporal domain of gait, while visual cues act in the spatial domain of gait. This study investigated how synchronizing steps with visual and auditory cues that are presented with different temporal structures could affect gait variability during treadmill walking. Three different temporal structured stimuli were used, invariant, fractal and random, in both modalities. Stride times, length and speed were determined, and their fractal scaling (an indicator of complexity) and coefficient of variation (CV) were calculated. No differences were observed in the CV, regardless of the cueing modality and the temporal structure of the stimuli. In terms of the stride time’s fractal scaling, we observed that the fractal stimulus induced higher values compared to random and invariant stimuli. The same was also observed in stride length, but only for the visual cueing modality. No differences were observed for stride speed. The selection of the cueing modality seems to be an important feature of gait rehabilitation. Visual cues are possibly a better choice due to the dependency on vision during walking. This is particularly evident during treadmill walking, a common practice in a clinical setting. Because of the treadmill effect on the temporal domain of gait, the use of auditory cues can be minimal, compared to visual cues.

## Introduction

The synchronization of walking to external cues has long been a matter for research, mainly due to its potential clinical application (del Olmo and Cudeiro, 2005; Spaulding et al., 2013; Hollands et al., 2015; van Ooijen et al., 2016). Typically, patients are instructed to synchronize their steps to auditory or visual cues while walking. These cues are usually presented at an invariant temporal or spatial fashion as is the case with a fixed tempo metronome or fixed-distance taped horizontal bars placed on the floor. The eventual goal is to improve the walking of these patients by reducing their abnormally increased variability of their spatiotemporal parameters, which has been associated with pathology (Hausdorff et al., 2001; Brach et al., 2007; Hamacher et al., 2011). Improvements in gait have been reported following the use of this invariant cueing technique (Pelton et al., 2010; Spaulding et al., 2013; Wittwer et al., 2013; Ghai et al., 2018). However, this approach disregards the natural stride-to-stride variations or fluctuations observed in healthy gait (Hausdorff et al., 1996). Such natural, healthy fluctuations are a fundamental characteristic of human movement, although increases or decreases of these fluctuations beyond their health levels represent a pathological system (Stergiou et al., 2006).

Numerous studies in the past two decades have shown that the dynamics of diverse biological signals examined over time (e.g., heart rate, gait and locomotor activity, neuronal activity) reveal the presence of physiological complexity in healthy systems, while a loss of complexity is present with aging and across a range of illnesses (Peng et al., 1995; Goldberger, 1996, 2001; Goldberger et al., 2002; Hausdorff et al., 1997, Hausdorff, 2007; Ashkenazy et al., 2002; Buzzi et al., 2003; Hu et al., 2004, 2007; Cavanaugh et al., 2005, 2010; Ivanov et al., 2007, 2009; Deffeyes et al., 2009; Decker et al., 2011; Smith et al., 2011; Bystritsky et al., 2012). The temporal ordering of the variations present in healthy individuals is characterized by scale invariant/fractal patterns, i.e., the temporal structure and statistical properties of inter-interval fluctuations remain similar over a wide range of time scales. The classic definition of a fractal, first described by Mandlebrot (1977), is a geometric object with “self-similarity” over multiple measurement scales (Lipsitz, 2002). Fractal patterns observed in biological signals such as heart rate (Costa et al., 2002), respiration (Peng et al., 2002) and walking strides (Hausdorff et al., 1996) measured over time, indicate that the time intervals between events are not equal, nor are they independent. Rather, there is a relationship between these variable intervals that holds beyond consecutive intervals, extending far forward and backward in time. The structure of these multi-scale interactions is therefore ordered yet flexible, relating to the concept in non-linear dynamics known as “complexity” (Stergiou et al., 2006; Stergiou and Decker, 2011).

Older adults and clinical populations have been shown to exhibit a breakdown of this complexity in their stride-to-stride fluctuations (Hausdorff et al., 1997, 1998; Kaipust et al., 2013). Similar findings have also been observed in healthy young adults walking to an invariant cueing paradigm (Terrier, 2012, 2016; Terrier and Dériaz, 2012; Kaipust et al., 2013; Hunt et al., 2014; Marmelat et al., 2014; Vaz et al., 2019). Several studies have shown that the manipulation of the temporal structure of the cueing provided can modulate stride-to-stride fluctuations (Kaipust et al., 2013; Hunt et al., 2014; Marmelat et al., 2014; Rhea et al., 2014; Vaz et al., 2019). The major finding of these studies was that when healthy young adults synchronized their steps to a random or an invariant cueing, their stride-to-stride fluctuations were altered toward patterns typically observed in older adults or clinical populations (Hausdorff et al., 1997). However, no changes were observed when they walked to fractal-structured cues, suggesting that this type of temporal structure does not affect the natural healthy fluctuations that exist in their walking patterns. Noteworthy are the findings from Hove et al. (2012) and Kaipust et al. (2013). These studies showed that older adults and individuals with Parkinson’s disease can change their stride-to-stride fluctuations toward those found in healthy young adults when they are asked to walk to this fractal-like pattern. This suggests that incorporating fractal-like fluctuations within the cueing modality can potentially lead to greater improvements in gait-related outcomes.

Although extensive research has shown that humans synchronize well with auditory cueing (Repp and Penel, 2002; Hove et al., 2013a, b), visual cueing could provide a different way to modulate gait. Visual cueing could mostly affect spatial, while auditory could mostly affect temporal gait parameters (Bertram and Ruina, 2001). Indeed, the relationship between gait spatiotemporal parameters has previously been shown to be constraint-dependent (Bertram and Ruina, 2001). Bertram and Ruina (2001) showed that for different fixed distances between visual cues, the relationship between stride time and stride speed is only marginally affected. However, when the constraint is set on the stride time (auditory cueing) or on stride speed via a constant-speed treadmill, stride time and stride speed increase as a function of frequency of the auditory cueing or the treadmill, respectively. The authors further suggested that the results in the stride length constraint condition may be due to “unnatural” walking, with subjects focusing on each foot placement. In other words, constraining stride length with visual spatial cues seems to lead to the allocation of higher levels of attention. Interestingly, it has been previously suggested that while walking to visual cues, vision is used to guide the steps providing a stronger reference for gait control (Hollands et al., 1995; Chapman and Hollands, 2010; Bank et al., 2011). Indeed, visual cues have been shown to elicit improved step adjustments compared to auditory cues (Bank et al., 2011). Likewise, healthy older adults were faster in adapting to a change of cueing modality from auditory to visual rather than vice versa, suggesting visual cues to be more effective in triggering gait adjustments (Bank et al., 2011).

The present study investigated the effects of different cueing modalities on the fluctuations of spatiotemporal gait parameters. Two different cueing modalities were examined, visual and auditory. For the visual modality, we used a virtual reality environment to provide spatial cues to direct the subject *where* to step. For the auditory modality, we provided auditory cues through speakers to direct the subject *when* to step. For both modalities we used three different structured conditions: invariant, fractal, and random. Due to the differences in attention allocation in visual compared to auditory cues, we hypothesized that both gait parameters will be greatly altered in visual cueing, particularly for stride length. Based on previous findings, we also hypothesize that stride speed will remain unaltered regardless of the cueing modality. We also hypothesized that stride time and length would exhibit changes in the complexity of their fluctuations according to the structure of the stimuli provided. For example, if a fractal-like stimulus was provided during auditory cueing, stride length would also exhibit fractal-like patterns.

## Materials and Methods

### Participants

Eighteen healthy young adults participated in this study. The subjects were randomly and equally assigned to an auditory (AUD; 26.3 ± 5.6 years, 1.76 ± 0.12 m, 75.8 ± 12.1 kg) or a visual (VIS; 25.4 ± 4.2 years, 1.78 ± 0.12 m, 84.1 ± 17.9 kg) group. The study was approved by the University of Nebraska Medical Center Institutional Review Board, and the study was carried out in accordance with the approved guidelines. Each participant provided informed consent before participation.

### Experimental Procedures

**FIGURE 1:**
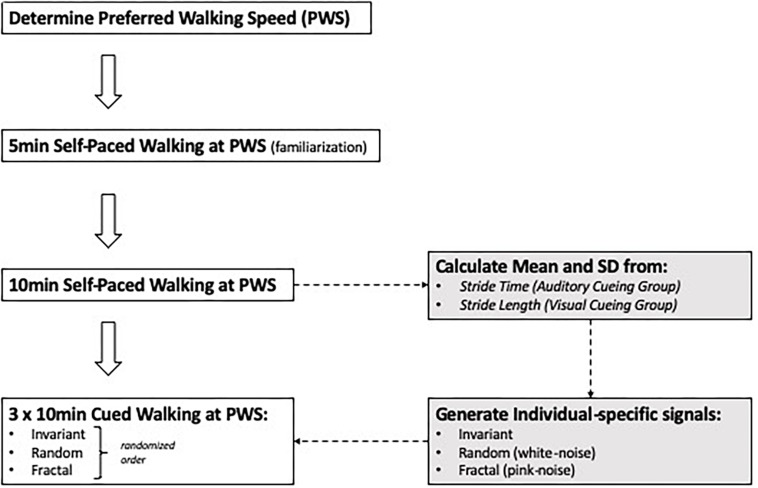
Schematic representation of the experimental protocol.

**FIGURE 2:**
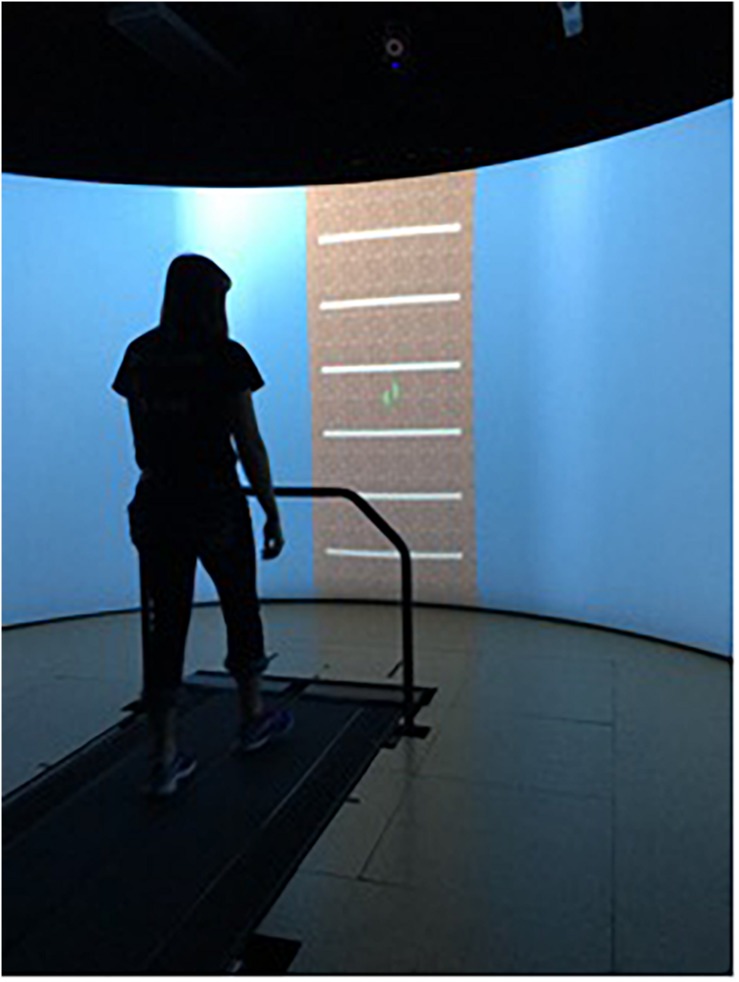
The visual cueing apparatus used. The horizontal bars represent the spatial cues where the subjects were instructed to step. Virtual footsteps were visible throughout the gait cycle.

Subjects were asked to complete four, 10-min walking trials on an instrumented treadmill (Bertec Corporation, Columbus, OH, United States). Before the first trial, the preferred walking speed (PWS) was determined for each subject (Jordan et al., 2007). For this, subjects were asked to start walking on the treadmill and indicate when comfortable with the treadmill’s speed, while the treadmill speed was increased in increments of 0.1 m/s. After the subjects mentioned that they were comfortable with the treadmill’s speed, it was increased further in increments of 0.01 m/s until the subjects indicated it was becoming “too fast to be comfortable.” The previous speed was set as their PWS. After the PWS was determined, a 5-min familiarization trial was conducted. Then, a 10-min trial was performed that was free of external cueing so that the average stride time (AUD cueing) or stride length (VIS cueing) could be determined and embedded in the cueing signals. The order of the three cueing conditions was randomized: invariant (INV), fractal (FRT) and random (RND) and a minimum of 10 min resting period was taken between conditions. The INV signal was generated using each subject’s mean PWS stride time or stride length and a standard deviation of zero. The FRT signal was generated using an approximation of a 10 dB/decade filter with a weighted sum of first-order filters (i.e., pink-noise), and RND was generated using a normal distribution of random numbers (i.e., white-noise). Each subject’s stride time (AUD group) or stride length (VIS group) and the standard deviation determined during the no cueing condition, were incorporated in the signal. Figure 1 illustrates the different steps of the present experimental protocol. The auditory cueing was provided through speakers as a single beat per stride, and the visual cueing was provided through the projection of horizontal bars on the screen, so the bars were traveling from the top of the screen toward the bottom (GRAIL, Motek) at the speed of the treadmill. For the latter modality, virtual footsteps were visible during the task on the screen (Figure 2), and the subjects were instructed to step on the horizontal bar with their virtual right foot. All the participants had prior experience in treadmill walking.

**FIGURE 3:**
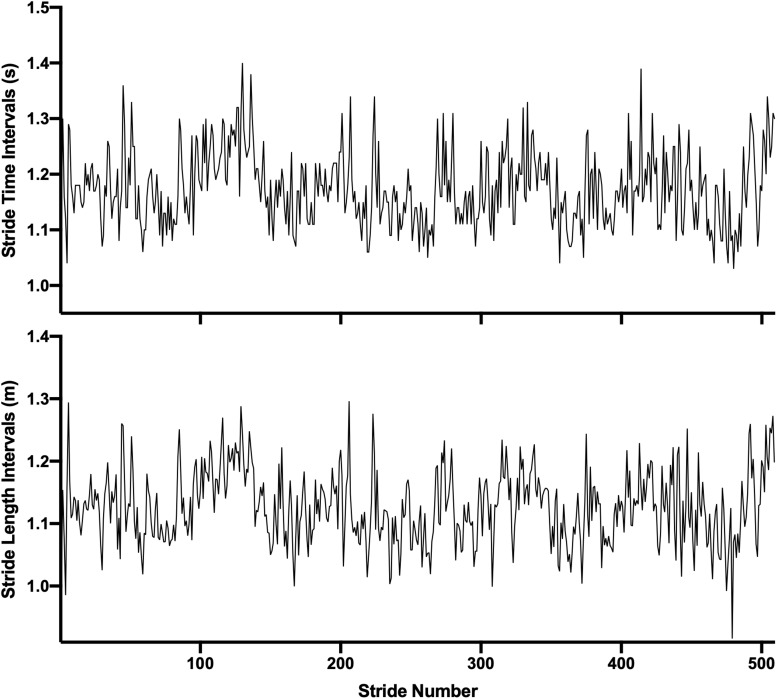
Example of stride time **(Upper)** and stride length **(Lower)** intervals time series from one subject while walking to the visual cues on the fractal condition.

Eight infrared cameras (Vicon Motion Systems Ltd., Oxford, United Kingdom) were used to detect the position of the retroreflective markers at 100 Hz. The markers were placed according to the Lower Limb Plug-in Gait Model (anterior iliac crests, posterior iliac crests, sacrum, knee lateral epicondyles, lateral thigh, lateral malleoli, shank, second metatarsal heads, calcaneus). Heel strikes were determined according to the Coordinate-Based Algorithm presented by Zeni et al. (2008). This method compares the anterior-posterior position of the heel and toe markers with the estimated center of mass (mean anterior-posterior position of the hip markers). The point at which the heel is at the greatest distance in front of the center of mass represents heel strike and the point where the toe is at the greatest distance behind the center of mass is toe-off. Stride time (ST) and stride length (SL) were then determined from the heel strike events (Figure 3). Stride speed (SS) was calculated as SL/ST.

### Data Analysis

**FIGURE 4:**
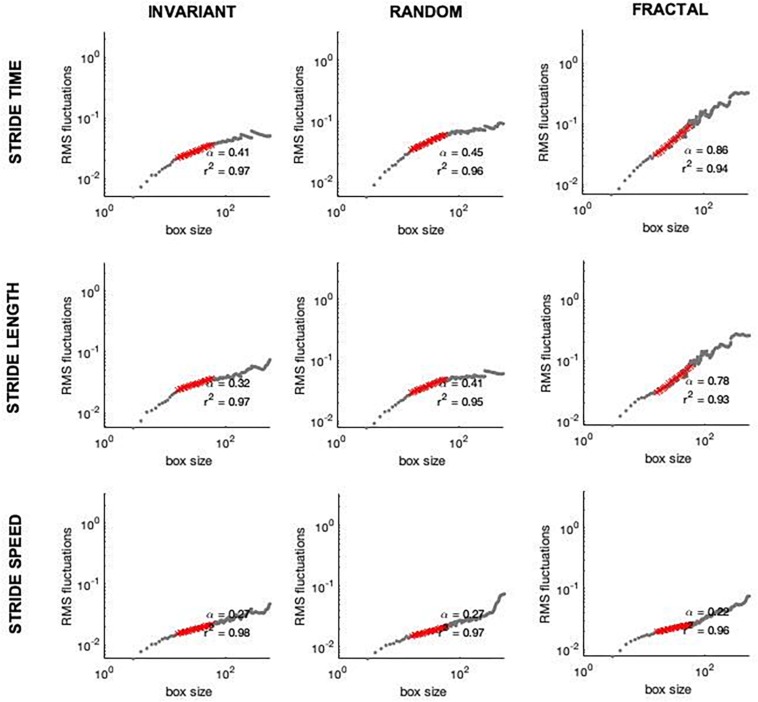
Examples of DFA plots on stride time **(Upper row)**, stride length **(Middle row)**, and stride speed **(Lower row)** from one subject while walking to invariant (first column), random (second column), and fractal (third column) cues.

The first 30 s of each trial were discarded to avoid any transient effects related to familiarization. The coefficient of variation (CV), a measure of amount in terms of variability, was calculated from each time series (ST, SL, and SS). The fractal scaling exponent, α, was also calculated using Detrended Fluctuation Analysis (DFA) from each time series (ST, SL, and SS; Figure 4). These parameters were all calculated from the time series from the right side. The fractal scaling exponent, α, quantifies the complexity of a physiological signal and detects the presence of statistical persistence in a given time series. Briefly in DFA, the integrated time series is divided into window sizes of length *n*. A least squares fit line is fit to the data in each window and data is detrended by subtracting the integrated time series from the least squares fit line. The root mean square is then calculated for each window and summed for the entire time series, F(n). The process is repeated with smaller and smaller n window sizes. Finally, the log F(n) is plotted against the log n, i.e., the root mean square versus the window sizes. The slope of this plot is the reported α-scaling value. If the α is greater than 0.5, the time series exhibits statistical persistence, indicating that increases are followed by increases and decreases are followed by decreases. If the α is smaller than 0.5, the time series is said to present anti-persistence, meaning increases are followed by decreases and vice versa. If the α is greater than 1, the time series is regarded as Brown noise (Jordan et al., 2007). Window sizes of 16 to N/9 were used for the analysis (Damouras et al., 2010), where N represents the data length. The Supplementary Material presents a more in-depth analysis that supports the existence of long-range dependence.

### Statistical Analysis

A two-way repeated measures ANOVA (group × stimulus) was used to evaluate the differences present in the CV and α-scaling exponent of the stride time (α-ST), stride length (α-SL) and stride speed (α-SS) time series. Mauchly’s test was implemented to test sphericity, and Greenhouse–Geisser correction was used when not verified. *Post hoc* analyses with Tukey’s tests were used to highlight specific differences between conditions. The alpha level was set at 0.05. Statistical analyses were performed using SPSS software (SPSS Inc., Chicago, IL, United States).

## Results

No differences between groups were observed for age, body mass and height. Table 1 presents the means and standard deviation from all gait parameters.

**TABLE 1:** Fractal scaling (α) and coefficient of variation (%) of the gait spatiotemporal parameters in each stimulus (Invariant, Fractal, and Random) and cueing modality (VIS – Visual; AUD – Auditory).

	Invariant		Fractal		Random	
	AUD	VIS	AUD	VIS	AUD	VIS
**Fractal scaling (α)**						
Stride time	0.53 ± 0.37	0.43 ± 0.14	0.78 ± 0.15	0.79 ± 0.08	0.70 ± 0.14	0.60 ± 0.09
Stride length	0.59 ± 0.26	0.23 ± 0.09	0.79 ± 0.15	0.72 ± 0.05	0.68 ± 0.11	0.58 ± 0.07
Stride speed	0.34 ± 0.06	0.42 ± 0.16	0.41 ± 0.09	0.35 ± 0.08	0.34 ± 0.09	0.34 ± 0.14
**Coefficient of variation (CV)**						
Stride time	4.48 ± 2.92	2.05 ± 0.52	5.38 ± 4.99	3.38 ± 1.26	4.51 ± 3.80	2.66 ± 1.03
Stride length	4.67 ± 2.48	2.19 ± 0.45	5.03 ± 4.35	3.40 ± 1.02	4.29 ± 3.41	2.86 ± 1.15
Stride speed	2.91 ± 1.18	2.23 ± 0.48	2.82 ± 1.66	2.62 ± 0.75	2.93 ± 1.83	2.48 ± 0.78

### Stride Time

No interaction effect was observed [*F*(1.165,18.633) = 0.107, *p* = 0.786, η^2^ = 0.007]. Additionally, no main effect for stimulus [*F*(1.165,18.633) = 1.360, *p* = 0.271, η^2^ = 0.078] nor group [*F*(1,16) = 3,489, *p* = 0.080, η^2^ = 0.179] was observed for the CV of the ST time series.

**FIGURE 5:**
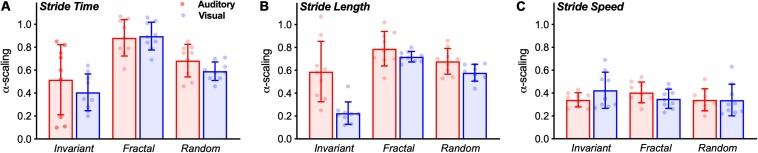
Bar graphs (Mean ± SD) with group results of the α-scaling of the stride time **(A)**, stride length **(B)**, and stride speed **(C)**. The dots represent individual values of each subject.

In terms of α-ST, no interaction effect was observed [*F*(2,32) = 0.744, *p* = 0.483, η^2^ = 0.044]. A significant main effect of stimulus was observed [*F*(2,32) = 16.672, *p* < 0.001, η^2^ = 0.510]. Pairwise comparison showed differences between all stimuli (Figure 5A). FRT was significantly higher than INV (*p* < 0.001) and RND (*p* = 0.005), and RND was significantly higher than INV (*p* = 0.024). No main effect for group was observed [*F*(1,16) = 1.007, *p* = 0.331, η^2^ = 0.059].

### Stride Length

No interaction effect was observed [*F*(1.251,20.014) = 0.521, *p* = 0.518, η^2^ = 0.032]. In addition, no main effect of stimulus [*F*(1.251,20.014) = 1.175, *p* = 0.305, η^2^ = 0.068] nor group [*F*(1,16) = 3.208, *p* = 0.092, η^2^ = 0.167] was observed for the CV of SL time series.

In terms of α-SL, an interaction effect was observed [*F*(2,32) = 7.648, *p* = 0.002, η^2^ = 0.323]. Additional one-way ANOVAs revealed a main effect for stimulus only in the VIS group [*F*(2,16) = 95.936, *p* < 0.001, η^2^ = 0.923]. Pairwise comparisons revealed that FRT was significantly higher than INV (*p* < 0.001) and RND (*p* = 0.008); and RND was higher than INV (*p* < 0.001).

### Stride Speed

No interaction effect was observed [*F*(1.198,19.166) = 0.364, *p* = 0.698, η^2^ = 0.022]. Additionally, no main effect of stimulus [*F*(1.198,19.166) = 0.000, *p* = 1.000, η^2^ = 0.000] nor for group [*F*(1,16) = 1.562, *p* = 0.229, η^2^ = 0.089] was observed for the CV of the SS time series.

Similarly, no interaction effect was observed [*F*(2,32) = 0.134, *p* = 0.698, η^2^ = 0.118]. No main effect of stimulus [*F*(2,32) = 0.956, *p* = 395, η^2^ = 0.056] nor for group [*F*(1,16) = 0.063, *p* = 0.806, η^2^ = 0.004] was observed for the α-SS (Figure 5C).

## Discussion

The present study investigated the effects of different cueing modalities (visual and auditory) on the fluctuations of spatiotemporal parameters during gait, when stimuli with different temporal structures were employed.

First, we hypothesized that the complexity of stride time and length would be greatly affected by visual cueing compared to auditory, while stride speed would not differ between cueing modalities. The present study results did not fully support this hypothesis. Specifically, we have not observed differences between cueing modalities. In addition, we calculated the CV of stride time, stride length and stride speed time series to assess the magnitude of the fluctuations. Contrary to Terrier’s results (Terrier, 2016), we did not observe differences in the CV across cueing modalities (referred as fluctuation magnitude in Terrier’s). Terrier discussed that these findings, showing greater CV in all spatiotemporal parameters while walking to visual cues, indicate that it was more challenging to walk with visual cues as compared to auditory. Our contradictory results may be due to the type of visual cues provided. Our visual cues were projected onto a screen in front of the subject, whereas in the former study the cues were projected onto the treadmill belt. Looking down while walking has previously been shown to affect energy expenditure (Wezenberg et al., 2011) and attentional cost (Peper et al., 2012), and to increase body motion compared to walking while looking straight ahead (Goodworth et al., 2015). Taken together, the potential impact of looking down to step on the cues might explain the different results observed by Terrier compared to our results. However, our visual cueing task and Terrier’s are substantially different in their nature. In our study, the subjects adjusted virtual steps that represented the subject’s feet on the screen; while in Terrier’s study, the subjects adjusted their actual feet on targets presented on the treadmill’s belt. These two forms of visual cueing may lead to different cognitive processing requirements. Regardless of the nature of the task, our results support the idea that the observed changes in the complexity of spatiotemporal gait parameters were not due to the magnitude of the fluctuations.

Secondly, we hypothesized that stride time and length would exhibit changes in the complexity of their fluctuations according to the structure of the stimuli provided regardless of the cueing modality. Overall, our results are generally in accordance with previous studies that showed a decrease in the complexity of gait when walking to a random or invariant stimulus toward values below 0.5 (anti-persistence) and an increased complexity when walking to a fractal stimulus, as commonly observed in healthy young adults walking with no cueing (Hunt et al., 2014; Marmelat et al., 2014; Vaz et al., 2019). In the present study, this was the case for stride time, i.e., stride time complexity followed the complexity of the type of stimulus provided (invariant, random or fractal), regardless of the cueing modality. However, in the case of stride length, this was only observed in the visual modality. No differences between stimuli was observed in auditory cueing (Figure 5). In addition, we have also found no differences between stimuli in the magnitude of the fluctuations. Overall, these results fit within the Optimal Movement Variability model (Stergiou et al., 2006), which suggests that a healthy system exhibits complexity and has an optimal level of variability. In the present study, we have used the α-scaling parameter to measure statistical persistence in a time series, which indicates complexity. An α-scaling around 0.8–1.0 is expected in human healthy walking. A decrease in this α-scaling represents a loss of complexity. Our fractal-like stimulus was able to maintain the gait complexity as commonly observed in healthy human uncued walking. Although we have not incorporated the uncued condition within our hypotheses and, hence, statistical design, it is important to note that extensive research has previously shown that the α-scaling in healthy young adults is ∼0.8–1.0 (Terrier and Dériaz, 2012; Hunt et al., 2014; Marmelat et al., 2014; Rhea et al., 2014; Vaz et al., 2019). This makes us confident of our results.

It is important to note that the treadmill has been shown to act as a metronome by decreasing the complexity of stride speed (Frenkel-Toledo et al., 2005). It has also been suggested that it requires a tighter control of stride speed (speed goal), while stride time and length can flexibly fluctuate to achieve the required speed. Theoretically, when a second goal is added (cueing), the regulation of spatiotemporal parameters is altered according to the cueing modality. Our results suggest that there be a trade-off between goals (constant-speed and synchronizing with the cues). When exposed to a double-task paradigm (e.g., constant-speed and stepping on cues), the participants either try to solve both tasks at the same time and constantly change gait control strategies or focus more on one of the tasks. In the present study, when following auditory cues on a treadmill, the subjects are likely to have selected the speed goal as a priority and compromised synchronization performance. Alternatively, some of them simply ignored or did not follow the cues. This is evidenced by the range of α-scaling exponent values observed in the auditory group. Although we were technically unable to determine the time difference between steps and cues, it is clear that some subjects showed a α-scaling higher than expected (Figure 5), suggesting that the synchronization with the stimulus was compromised and the fluctuation pattern was not followed by some of the subjects. For the visual cueing, however, this was not observed. The α-scaling was shown to be in accordance to the expected complexity level (note the group’s standard deviation of stride length for the visual group compared to the stride time for the auditory group – Figure 5), indicating that the subjects followed the cues with greater accuracy.

There are several possible interpretations for the differences found in the visual that were not observed in the auditory group (i.e., stride length complexity). First, the visual cueing has a direct effect on a spatial feature of human gait, compared to the auditory that acts preferentially in the temporal domain. Therefore, the effect of the treadmill in the temporal domain is not as conflicting in the visual cueing modality as it is in the auditory. Furthermore, the visual cues were designed based on stride length, while auditory cues were designed based on stride time. Second, the visual cues provided the participant with feedback and feedforward information while in the auditory cueing no feedforward information was available. Lastly, the visual cues were presented in a continuous fashion, i.e., the participant had constant information about his/her location in relation to the horizontal bars and was aware of the moving bar ahead of time. On the other hand, the auditory cues were presented in a discrete format. This would play in favor of the visual cueing modality by allowing the participant to plan ahead of time to step on the horizontal bar. A possible alternative to effectively manipulate stride time complexity would be to provide a continuous visual temporal stimulus, such as a moving bar. The decision whether a patient would benefit more from a temporal or a spatial stimulus depends on a proper clinical gait assessment and an understanding of the pathology. For example, Stroke survivors have been shown to benefit from spatial stimulus (Hollands et al., 2015), while patients with Parkinson’s disease may benefit more from temporal stimuli (Ghai et al., 2018). However, a visual stimulus could be preferred due to the possibility of providing feedforward and feedback information during gait rehabilitation, compared to auditory stimuli.

Although it is possible that a greater sample size would have resulted in similar findings in the auditory cueing compared to the visual, particularly in terms of stride length complexity, the present study’s results do suggest that overall humans may engage more with a visual continuous stimulus. We would also like to point out that even though the sample size is indeed small, our effects sizes were high, particularly in the interaction effect in stride length complexity. Whether the same engagement would have been obtained if an auditory continuous stimulus had been used is a matter that should be further studied when walking synchronized to external cues. Future studies in this area should also test whether the present findings are the result of the sensory system that receives the stimulus (auditory or visual) or the nature of the stimulus. Developing an auditory continuous stimulus (e.g., through variation of volume) could be a possible interesting alternative to test this hypothesis. This will still allow the comparison between a spatial visual versus an auditory temporal stimulus, but where both provide continuous feedback and feedforward information to the user.

## Conclusion

The nature of the cueing modality is an important feature of synchronized walking. The present study results suggest that spatial cues require an increased attentional allocation that results in an improved synchronization. In addition, temporal cues during treadmill walking seem to be conflicting as the treadmill is known to have a similar effect as auditory temporal cueing. On the other hand, spatial cues act in the spatial domain of gait and the system is able to deal with the dual-task goal (treadmill and spatial cues). This conflict during auditory cueing is likely the major cause of the differences between spatial and temporal cues observed in the present study. These findings are of major interest since gait rehabilitation protocols extensively use treadmills to guarantee safety and supervision throughout the walking sessions. Therefore, those that commonly use auditory temporal cues in rehabilitation should re-think and consider using spatial cues, for the benefit of the individual. The use of a stimulus that presents a fractal-like structure was also shown to lead to patterns of gait complexity, commonly observed during uncued walking in healthy young adults, particularly evident while synchronizing walking with visual cues.

## Data Availability Statement

The datasets generated for this study are available on request to the corresponding author.

## Ethics Statement

The studies involving human participants were reviewed and approved by the Institutional Review Board of the University of Nebraska Medical Center. The patients/participants provided their written informed consent to participate in this study.

## Author Contributions

MM, TR, JF-H, and NS designed the project. TR and JF-H developed the stimuli setup and performed the experiments. JV and TR analyzed the data. JV, MM, and NS interpreted the results. JV wrote the first draft of the manuscript. All authors read and approved the final version of the manuscript.

## Conflict of Interest

The authors declare that the research was conducted in the absence of any commercial or financial relationships that could be construed as a potential conflict of interest.
