# Supplementary material for: Auditory and Visual External Cues Have Different Effects on Spatial but Similar Effects on Temporal Measures of Gait Variability
Source: Front Physiol. 2020 Feb 11;11:67. doi: 10.3389/fphys.2020.00067 (PMC7026509; doi:10.3389/fphys.2020.00067)
Supplement: Supplementary file 1 [file Presentation_1.pdf]

Supplementary Material

Auditory and visual external cues have different effects on spatial but similar effects on temporal measures of gait variability

Vaz, J.R.[1,2]; Rand, T.J.[1,3]; Fujan-Hansen, J.[1], Mukherjee, M.[1], Stergiou, N.[1,4]

[1] Department of Biomechanics, University of Nebraska at Omaha, Omaha, NE, USA

[2] CIPER, Faculty of Human Kinetics, University of Lisbon, Portugal

[3] The Paley Institute, West Palm Beach, FL, USA

[4] Department of Environmental Agricultural and Occupational Health, University of Nebraska Medical Center, Omaha, NE, USA

The purpose of this supplementary material was to provide the readers with a more in-depth analysis that objectively assessed the presence of long-range dependence and, hence, justifying the use of detrended fluctuations analysis (DFA). Here we present examples from the signals presented on Figure 3 from the manuscript.

First, we analyzed the power spectrums and observed the presence of a few greater peaks (Figure S1). Thus, one could argue that the observed results could have resulted from the superposition of few mainly dominant frequencies. Although the power spectrums globally appeared to be scale invariant, we have conducted an objective analysis by means of a more in-depth statistical analysis to investigate the presence of long-range dependence: ARFIMA modelling (Wagenmakers et al., 2004; 2005; Torre et al., 2007). In order to test the presence of long-range dependence, we ran the following nine ARFIMA(q,d,p) models: ARFIMA(0,0,0), ARFIMA(1,0,0), ARFIMA(2,0,0), ARFIMA(0,0,1), ARFIMA(1,0,1), ARFIMA(2,0,1), ARFIMA(0,0,2), ARFIMA(1,0,2), ARFIMA(2,0,2). Additionally, we also tested the corresponding ARMA models to further investigate if the signals better fitted to an ARFIMA (long-range dependence) compared to an ARMA model (short-range dependence). Tables below (S1 and S2) present the models' weights Bayes Information Criterion (BIC) and *p*-values. The transformation of the BIC values to weights was performed to allow a better interpretation and identification of the best model. This was conducted according to Wagenmakers and Farrell (2004). Moreover, and according to Torre et al. (2007) two criteria are proposed for detecting the presence of long-range dependence in the time series: "1) the best model (i.e. the model with the largest weight) should be an ARFIMA (p,d,q), d being significantly different from 0; and (2) the sum of the weights of the ARFIMA models should be higher than the sum of the weights of the ARMA models". The present analysis showed that: 1) the best model was an ARFIMA model and 2) the sum of the ARFIMA models' weights represents greater probability of ARFIMA models overcoming ARMA counterparts. For the signals under investigation, the sum of the weights of the ARFIMA models

represents 100%. The robust ARFIMA modelling analysis indicates that these time series present long-range dependence.

Figure S1. Power spectrum density for Stride Time (left) and Stride Length (right) series.

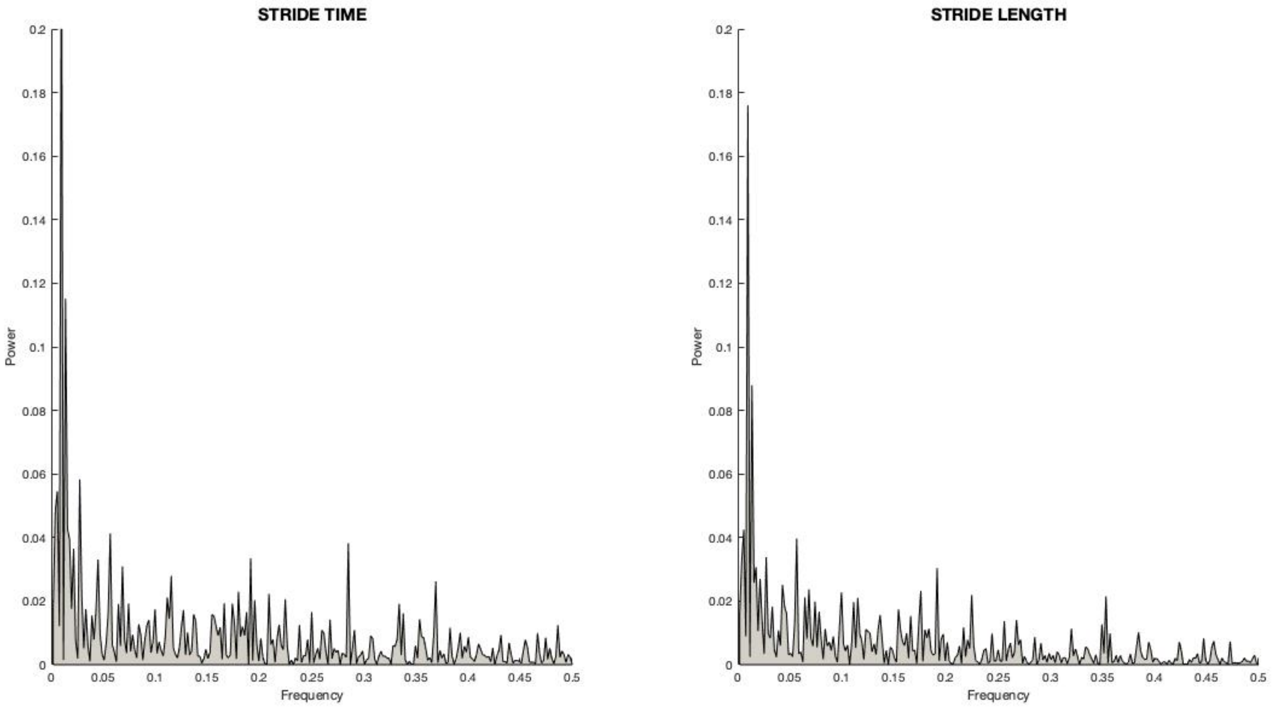

Table S1. *ARMA/ARFIMA results from the stride time signal on Figure 3 (upper panel); The best model is highlighted in grey and bold.*

ARMA			ARFIMA		
Model (*p,q*)	ω_i BIC	*p-value*	Model (*p,d,q*)	ω_iBIC	*p-value*
(0,0)	0.000	0.001	**(0,0,0)**	**0.684**	**< 0.001**
(1,0)	0.000	0.003	(1,0,0)	0.106	< 0.001
(2,0)	0.000	0.018	(2,0,0)	0.018	< 0.001
(0,1)	0.000	0.001	(0,0,1)	0.157	< 0.001
(1,1)	0.000	0.080	(1,0,1)	0.015	< 0.001
(2,1)	0.000	0.817	(2,0,1)	0.001	< 0.001
(0,2)	0.000	0.001	(0,0,2)	0.019	< 0.001
(1,2)	0.000	0.932	(1,0,2)	0.001	< 0.001
(2,2)	0.000	0.938	(2,0,2)	0.000	< 0.001

Table *S2.* *ARMA/ARFIMA results from the stride length signal on Figure 3 (lower panel); The best model is highlighted in grey and bold.*

ARMA			ARFIMA		
Model (*p,q*)	ω_iBIC	*p-value*	Model (*p,d,q*)	ω_iBIC	*p-value*
(0,0)	0.000	0.001	(0,0,0)	0.006	< 0.001
(1,0)	0.000	0.055	(1,0,0)	0.115	0.001
(2,0)	0.000	0.057	(2,0,0)	0.038	< 0.001
(0,1)	0.000	0.001	**(0,0,1)**	**0.714**	**< 0.001**
(1,1)	0.000	0.059	(1,0,1)	0.048	< 0.001
(2,1)	0.000	0.097	(2,0,1)	0.003	< 0.001
(0,2)	0.000	0.001	(0,0,2)	0.046	< 0.001
(1,2)	0.000	0.846	(1,0,2)	0.030	0.724
(2,2)	0.000	0.855	(2,0,2)	0.000	0.015

References

Torre, K., Delignières, D., and Lemoine, L. (2007). Detection of long-range dependence and estimation of fractal exponents through ARFIMA modelling. *Br J Math Statistical Psychology* 60, 85–106. doi:10.1348/000711005x89513.

Wagenmakers, E.-J., and Farrell, S. (2004). AIC model selection using Akaike weights. *Psychon B Rev* 11, 192–196. doi:10.3758/bf03206482.

Wagenmakers, E.-J., Farrell, S., and Ratcliff, R. (2004). Estimation and interpretation of 1/f alpha noise in human cognition. *Psychon B Rev* 11, 579–615. doi:10.3758/bf03196615.

Wagenmakers, E.-J., Farrell, S., and Ratcliff, R. (2005). Human Cognition and a Pile of Sand: A Discussion on Serial Correlations and Self-Organized Criticality. *J Exp Psychology Gen* 134, 108–116. doi:10.1037/0096-3445.134.1.108.
